# Biochemical and growth responses of silver maple (*Acer saccharinum* L.) to sodium chloride and calcium chloride

**DOI:** 10.7717/peerj.5958

**Published:** 2018-12-21

**Authors:** Jacek Patykowski, Jeremi Kołodziejek, Mateusz Wala

**Affiliations:** 1 Department of Plant Physiology and Biochemistry, Faculty of Biology and Environmental Protection, University of Lodz, Lodz, Poland; 2 Department of Geobotany and Plant Ecology, Faculty of Biology and Environmental Protection, University of Lodz, Lodz, Poland

**Keywords:** *Acer saccharinum* L., Sodium chloride, Calcium chloride, Salt stress, Plant growth, Proline, Antioxidant enzymes

## Abstract

The present research investigated the response of silver maple (*Acer saccharinum* L.) to salt treatment. The short- and long-term effects of NaCl and CaCl_2_ treatments on plant fitness characteristics (growth parameters, leaf chlorophyll content) and biochemical stress-coping mechanisms (proline accumulation as well as enzymatic activities) were examined. We found that the silver maple response to salt stress strictly depended on salt type and dose—calcium chloride was less toxic than sodium chloride, but high concentrations of both salts negatively influenced plant growth. The accumulation of proline, slight changes in the activity of superoxide dismutase and marked changes in catalase and peroxidase activities in the roots and leaves indicated complexity of the plant response. It was also shown that after one year, enzymatic parameters were restabilized, which indicates plant recovery, but the reduced mass of seedlings suggests that one year is not enough to cope with the prolonged cyclic salt stress, both resulting from NaCl and CaCl_2_ application. Therefore, seedlings of silver maple should be considered as moderately susceptible to salinity. Hence, it is recommended to use silver maple on non-de-iced urban areas, while planting on often de-iced roads should be avoided.

## Introduction

In urban areas, each winter, various chemical and abrasive materials are used on roads and sidewalks to prevent ice formation. The two most commonly used de-icing salts worldwide are sodium (Na^+^) chloride (NaCl) and calcium (Ca^2+^) chloride (CaCl_2_). Although CaCl_2_ is better for melting ice (Nixon, 2008) and less damaging to plants (Trajkova, Papadantonakis & Savvas, 2006), NaCl is used most extensively (c.a. 9–10 million tons per year compared to 0.3 million tons of calcium chloride—Ramakrishna & Viraraghavan, 2005) because it is less expensive and easier to handle (Nixon, 2008). The commonly used salts are dispensed directly or mixed with sand before they are applied to the road (Simini & Leone, 1986).

Salinity of soils located near de-iced roads changes with the distance from road margin. For example, it was indicated that Na^+^ concentration within five meters from road margin remains at constant concentration (101–154 mg kg^−1^) and then drastically decreased within next five meters (Bryson & Barker, 2002). The maximal salt accumulation zone is located about one meter from road margin where trees are often planted in urban environment (Cunningham et al., 2008). It is also worth noticing that salt accumulation depends on abiotic factors (e.g., soil properties, landscape, weather) as well as biotic factors (e.g., local vegetation) (Cunningham et al., 2008) and may show strong variation (Bryson & Barker, 2002; Cunningham et al., 2008). Therefore, in many cases, accurate estimation of salt concentration within the nearest area from de-iced roads is hard, but severe accumulation of salts in non-permeable soils is highly probable due to winter maintenance of roads. Although the application of salts is necessary for traffic safety, it can cause damage to adjacent roadside trees and shrubs. Several de-icing investigations have attributed roadside plant damage to the combination of aerial spray of road salts, direct foliar contact with salt ions and high soil salt concentrations (Davison, 1971; Dirr, 1976; Hofstra, Hall & Lumis, 1979; Czerniawska-Kusza, Kusza & Duzyński, 2004; Gałuszka et al., 2011; Douglas, 2011). It is established that at least along major highways, salt spray causes more damage to trees and shrubs than salt absorbed from the soil (Dirr, 1976; Hofstra, Hall & Lumis, 1979; Sucoff, Hong & Wood, 1976; Langille, 1976). Salt spray injury is more commonly observed in evergreen coniferous tree species, while soil uptake injury is more common in deciduous trees (Hofstra, Hall & Lumis, 1979; Mekdaschi et al., 1988; Kozłowski, 1997; Bryson & Barker, 2002). Salts affect plant growth in several ways. De-icing salts cause damage through direct contact of the salt solution with plant foliage (referred to as “spray zone” injury) and through chemical and physical modification of the soil as a result of salt accumulation (Douglas, 2011). Dissolved salt ions originating from chemical de-icers (e.g., Na^+^, Cl^−^) can cause osmotic stress in plants (Paul, Rocher & Impens, 1987). High concentrations of inorganic chloride salts in the soil make cations (such as potassium, calcium, and magnesium) unavailable to plant roots (White & Broadley, 2001; Tester & Davenport, 2003). Furthermore, the accumulation of specific ions can cause toxicity within plant cells (Levitt, 1980; White & Broadley, 2001; Raveh & Levy, 2005) and reduce both frost hardiness (Sucoff, Hong & Wood, 1976) and drought tolerance (Maas, 1985).

Salinity is a stress factor enhancing the production of reactive oxygen species (ROS) which can lead to oxidative damage in plant cells. Therefore, robust metabolism of ROS is believed to protect plant tissues from injuries under salt conditions as well as during other abiotic stresses (e.g., drought and light stress) (Miller et al., 2010). On the other hand, tuned ROS balance play role in signal transduction pathways and are among factors activating plant responses to environmental stimuli (Miller et al., 2010). This can be modulated and fine-tuned by enzymatic antioxidants, such as superoxide dismutase (SOD), catalase (CAT) and peroxidase (POD). Increasing SOD activity was shown to be involved in salt stress tolerance in herbaceous (reviewed by Gill & Tuteja, 2010) and woody plant species (Wang et al., 2010). A similar conclusion could be drawn with regard to studies on the involvement of CAT and POD in the response of *Populus cathayana* Rehder to salt stress (Yang et al., 2009). The accumulation of osmolytes is also one of the salt-induced stress coping mechanisms. During NaCl stress, Na^+^ ions are stored in vacuoles, while potassium ions (K^+^) and compatible chemicals (such as proline, sucrose, glycine, betaine, mannitol) are accumulated in the cytosol and other organelles to balance the osmotic pressure of ions in the vacuoles (Wang et al., 2005; Lee et al., 2007; Székely et al., 2008). Similar changes can be observed during cold (Naidu et al., 1991), drought (Choudhary, Sairam & Tyagi, 2005), oxidative (Yang, Lan & Gong, 2009) and heavy metal (Siripornadulsil et al., 2002) stress. Proline is probably the most widely distributed osmolyte, and it occurs not only in plants but also in many other organisms (Delauney & Verma, 1993). In addition to regulating osmotic pressure, proline is implicated in plant tissue defense against osmotic stress and in the protection of plasma membrane integrity (Mansour, 1998) or as a source of carbon and nitrogen (Peng, Lu & Verma, 1997; Soshinkova et al., 2013).

Silver maple (*Acer saccharinum* L.) is a common, floodplain deciduous tree originating from North America that is adapted to saturated soils and flooding (Saeki et al., 2011). It is also common and widely cultivated in the northern temperate zone (Day, Seiler & Persaud, 2000). In many countries, including Poland (where silver maple is not protected species), this tree species is planted in street settings, parks, and residential and commercial landscapes. Its widespread popularity results from its rapid growth and attractive appearance—leaves are gently-lobed, deeply dissected and glaucous silvery-white on abaxial side (Saeki et al., 2011). From a practical point of view, appropriate tree selection should be based on information about the relationship between tree growth and stress-coping mechanisms that pertain to the urban environment, such as salt stress, soil compaction and heat island effect. Although some studies have suggested that silver maple is a plant sensitive to salinity (Buschbom, 1968; Dmuchowski, Baczewska & Bra̧goszewska, 2011; Dmuchowski, Brogowski & Baczewska, 2011), the available information about the salinity tolerance level of silver maple at different stages of growth is scarce, e.g., the salinity tolerance of silver maple seedlings is not well known. Furthermore, studies on the role of antioxidant enzymes during salt stress and on the plant growth parameters accompanying it allow us to estimate range and amplitude of response to salinity in this woody species. Therefore, the aim of this investigation was to determine the effects of de-icing salts on the silver maple at four time-steps (14 d, 28 d, 180 d and 360 d). We also assayed how different salinity levels altered the biochemical stress-coping mechanism and changed the fitness of this widely used plant.

## Material and Methods

### Seed material

Mature seeds were collected from 10 randomly selected silver maple (*Acer saccharinum* L.) trees (c.a. 10% of total seed pool were gathered from single mother tree) in Lodz, central Poland (19°20′N/19°38′E), on 30 June 2013. The local climate is temperate, and the seasons are clearly differentiated. Meteorological data (Lodz Meteorological Station) based on a 10-year period (2000–2010) indicated that the mean annual temperature was 8.8 °C. The average low temperature during winter was ≤2.5 °C, and the average high temperature during summer was 22.4 °C. Annual total precipitation (rain and snow) was 587.2 mm. The frost-free period averages 271 d (days).

### Seed germination

The silver maple seeds were mixed before tests in order to fulfill the randomization requirement. The seeds were surface sterilized with 70% (v/v) ethanol for 1 min and with 10% (v/v) sodium hydrochloride for 20 min. Then, the sterilized seeds were soaked for 4 h (hours) in sterile distilled water. The seeds were germinated on trays (100 seeds per tray) containing wet perlite (previously tested not to release salt) at 25 °C. All used seeds were viable and the final germination rate was above 75%.

### Growth conditions

After a week, seedlings of similar size were individually transplanted into 500-cm^3^ pots filled with perlite. The pots were watered to saturation twice daily with Hoagland’s solution (half strength) (Hoagland & Arnon, 1950). The plants were grown in a growth chamber with a 16:8 h photoperiod and a light intensity of 900–1,200 µmol m^−2^ s^−1^. Relative humidity was maintained between 60% and 70% and thermoperiod of 25 °C/18 °C (day/night). The pots of replicate treatments (see below) were rotated periodically within the chamber rooms to reduce the effects of possible temperature and/or light variation.

### Plant treatments

After 2 months, the seedlings were divided into 12 groups. Each group was treated with one solution of NaCl (0, 10, 30, 60, 100 and 120 mM) or CaCl_2_ (0, 6.7, 20, 40, 66 and 80 mM). The control groups (0 NaCl and a 0 CaCl_2_) were not treated with any salt solution. The plants were treated three times at two-week intervals with 25 cm^3^ of the appropriate salt solutions. To prevent the accumulation of salts in perlite, distilled water was applied every three days. The concentrations of NaCl and CaCl_2_ were chosen to achieve the same concentration of dissociated ions in the corresponding solutions (10 mM NaCl and the corresponding 6.7 mM CaCl_2_, 120 mM NaCl and 80 mM corresponding CaCl_2_). NaCl and CaCl_2_ are used in Poland both is solid (10–30 g m^−2^ of NaCl) and dissolved (40–160  cm^3^ m^−2^ of 25% NaCl or 15/30% CaCl_2_ solution) form (Czarna, 2013). The concentrations of salt solutions used in this study were chosen to simulate distribution of road salt residues which depends on distance from salted surfaces (the maximal accumulation of salt can be observed in area located nearest to the road margin).

Then, 14 and 28 d after treatment (three doses of salt solution), leaves and roots were collected for the analysis of enzymatic and nonenzymatic parameters. These parameters were analyzed again 360 d after treatment. In addition, seedling growth was measured 180 and 360 d after treatment. For each treatment in each time point, four plants were subjected to biochemical analysis and another four plants were subjected to analysis of growth parameters.

### Growth parameters

Fresh (FW) and dry (DW) weights of the plant shoots and roots were measured at 180 and 360 d. To determine dry mass, the material was dried for 48 h in a forced-draft oven at 60 °C. To determine the relative growth rate (RGR), plants were harvested immediately prior to the beginning of the salt treatments. Thereafter, successive harvests were taken at 180 and 360 d. The relative growth rate (RGR) was calculated using the following formula: RGR = (ln mass^2^ * ln mass^1^/time) (Khan, Unagr & Showater, 2000). The dry mass of each harvest was used for calculations. RGRs were expressed in g g^−1^ FW d^−1^.

### Biochemical analysis

Chlorophyll *a* and *b* contents in the fresh leaf samples were measured using the method of Arnon (1949) and Ashraf et al. (1994). Pigment concentrations were calculated from the absorbance of extract at 663 nm (A663) and 645 nm (A656) using the following formula:

Chlorophyll *a* (mg g^−1^ FW) = [12.7 * (A663) − 2.69 * (A645)] * 0.5

Chlorophyll *b* (mg g^−1^ FW) = [22.9 * (A645) − 4.69 * (A663)] * 0.5

The content of photosynthetic pigments was expressed in mg g^−1^ FW.

Free proline accumulation was determined using the method of Bates, Waldren & Teare (1973). The content of free proline was expressed in mg g^−1^ FW. For enzymatic analysis, the leaf fragments were homogenized (1:10 ratio) in 0.05 phosphate buffer (pH = 7.0) containing 1mM EDTA and 1% soluble PVP (polyvinylpyrrolidone). Then, the homogenates were centrifuged at 15,000 rpm for 15 min at 4 °C. The resulting supernatants were immediately used to analyse protein content and enzymatic activity. Superoxide dismutase (SOD, EC 1.15.1.1) activity was determined spectrophotometrically using the assay of Beauchamp & Fridovich (1971). The absorbance was measured 10 min after starting at a wavelength of 560 nm. The total reaction mixture of 2.7 cm^3^ contained 50 mM potassium phosphate buffer pH 7.8, 13 mM methionine, 75 µM NBT, 2 µM riboflavin, 0.1 mM EDTA and the enzyme extract. The reaction was started by turning on the UV lamp. The enzyme activity was expressed in units, each representing the amount of the enzyme required to inhibit 50% of the photochemical reduction of NBT, min^−1^ mg^−1^ protein. Catalase (CAT, EC 1.11.1.6) activity was determined spectrophotometrically using the assay of Dhindsa, Plumb-Dhindsa & Thorpe (1981). Absorbance was measured at a wavelength of 240 nm (ε = 36.1 mM^−1^ cm^−1^). The total reaction mixture of 2 cm^3^ contained 50 mM potassium phosphate buffer pH 7.0, 15 mM H_2_O_2_ and the enzyme extract. The enzyme activity was expressed in units, each representing 1 mM H_2_O_2_ decomposed, min^−1^ mg^−1^ protein. Peroxidase (POD, EC 1.11.1.7) activity was determined spectrophotometrically using the assay of Maehly & Chance (1954). Absorbance was measured at a wavelength of 470 nm (ε = 26.6 mM^−1^ cm^−1^). The total reaction mixture of 2 cm^3^ contained 25 mM acetate buffer pH 5.6, 5 mM guaiacol, 15 mM H_2_O_2_ and the enzyme extract. The enzyme activity was expressed in units, each representing 1 µmol tetraguaiacol formed, min^−1^ mg^−1^ protein. Protein content was assayed by the Bradford method (1976) using bovine serum albumin as a standard.

### Statistical analysis

Measurements at each time point were obtained from four independent replicates. The data for all statistical tests were log_10_ transformed to meet the assumptions of normality and homogeneity of variances implicit in parametric statistical procedures. The data were analyzed by one-, two- or three-way ANOVA. When significant differences were found among means, Tukey’s multiple comparison post hoc test (HSD-test) after one-way ANOVA was carried out to determine if significant (*P* < 0.05) differences occurred between individual treatments. Statistical analysis was carried out using Statistica 10 PL.

### Ethics statement

The plant material (seeds of *Acer saccharinum* L.) was collected in Lodz. The area of Lodz city reported in this paper is controlled by the government of the Poland and is not privately owned, nor protected. The species studied here is not yet a protected species in Poland and permit for collection of seeds was not required (Poland Ministry of the Environment, 2013; Poland Ministry of the Environment, 2014). Furthermore, neither local population size nor population fitness was affected.

## Results

### Growth Parameters

In general, both salt types caused a reduction in the fresh weight of the seedlings; however, a greater reduction in the shoot and root fresh weight occurred after NaCl treatments (all corresponding NaCl vs CaCl_2_ comparisons in each time point were significant at *P* < 0.001). The total dry weight accumulation was not significantly inhibited at low salinities (<30 mM)—this parameter was strongly inhibited at concentrations >60 mM of NaCl and >40 mM of CaCl_2_ (Table 1). On the other hand, weight of seedlings showed substantial promotion at low salinity caused by CaCl_2_ (6.7 and 20 mM for both fresh and weight dry weight) after the first harvest (Table 1). Further increasing salinity caused a progressive decline in weight. Analysis of variance of the salt-treated plants indicated a greater reduction of the fresh weight of roots than of shoots. In the contrary, dry weight of shoots were much more affected by increasing salt concentration than dry weight of roots. (Table 1). Only dry weight of roots were not significantly affected by interaction of all studied factors, while other weight parameters were strongly affected by the salt type, dose and time as well as their interaction (Table S2).

**Table 1:** The fresh and dry weight of the roots and shoots from the silver maple seedlings exposed to different salt type (NaCl or CaCl_2_) and dose after 180 and 360 d.

Salinity (mM)	Fresh weight (mg plant^−1^)			Dry weight (mg plant^−1^)		
	root	shoot	total	root	shoot	total
After 180 d						
0 NaCl	480.6 ± 12.9^1^	310.2 ± 10.9^1^	790.8 ± 12.0^1^	62.7 ± 1.8^1^	214.5 ± 8.9^1^	277.2 ± 8.4^1^
10 NaCl	467.0 ± 8.9^1^	305.7 ± 5.3^1^	772.7 ± 21.6^1^	60.3 ± 5.4^1^	227.0 ± 11.3^1^	287.3 ± 5.7^1^
30 NaCl	322.4 ± 5.5^2^	258.4 ± 9.8^2^	580.8 ± 16.6^2^	56.1 ± 4.7^1^^2^	188.9 ± 6.8^2^	245.0 ± 8.1^2^
60 NaCl	309.1 ± 7.6^2^	242.7 ± 7.6^2^^3^	551.8 ± 12.8^2^	55.9 ± 8.9^1^^2^	155.0 ± 4.6^3^	210.9 ± 4.2^3^
100 NaCl	244.5 ± 12.2^3^	225.7 ± 12.1^3^	470.2 ± 8.9^3^	51.5 ± 2.8^1^^2^	123.2 ± 5.2^4^	174.7 ± 3.3^4^
120 NaCl	217.0 ± 15.0^4^	197.0 ± 5.8^4^	414.0 ± 9.8^4^	46.0 ± 6.3^2^	100.7 ± 6.1^5^	146.7 ± 4.1^5^
0 CaCl_2_	536.5 ± 4.8^3^	414.1 ± 13.5^2^	950.8 ± 12.8^2^	87.2 ± 5.2^2^	272.4 ± 6.3^1^	359.6 ± 5.7^2^
6.7 CaCl_2_	565.9 ± 6.5^2^	416.5 ± 6.5^2^	982.4 ± 21.8^2^	103.1 ± 5.7^1^	280.5 ± 7.5^1^	383.6 ± 6.3^1^
20 CaCl_2_	596.3 ± 7.4^1^	447.2 ± 7.9^1^	1043.5 ± 23.2^1^	108.9 ± 2.8^1^	236.6 ± 9.5^2^	345.5 ± 8.7^2^
40 CaCl_2_	499.0 ± 12.2^4^	385.3 ± 11.2^3^	884.3 ± 14.2^3^	81.6 ± 7.5^2^^3^	197.0 ± 6.8^3^	278.6 ± 7.6^3^
66 CaCl_2_	457.7 ± 8.6^5^	358.6 ± 8.1^4^	816.3 ± 15.9^4^	72.5 ± 4.6^3^	152.7 ± 6.3^4^	225.2 ± 3.5^4^
80 CaCl_2_	412.5 ± 8.6^6^	336.7 ± 6.8^5^	749.2 ± 11.6^5^	59.6 ± 4.6^4^	134.8 ± 5.7^5^	194.4 ± 4.2^5^
After 360 d						
0 NaCl	553.8 ± 12.9^1^	670.9 ± 10.4^2^	1224.7 ± 21.6^2^	86.4 ± 4.3^1^	407.1 ± 6.5^2^	493.5 ± 9.5^2^
10 NaCl	573.2 ± 8.9^1^	771.4 ± 27.3^1^	1344.6 ± 25.2^1^	81.5 ± 5.6^1^^2^	448.0 ± 6.3^1^	529.5 ± 6.8^1^
30 NaCl	412.6 ± 5.5^2^	607.5 ± 18.2^3^	1020.1 ± 19.7^3^	70.8 ± 7.1^2^	344.5 ± 11.8^3^	415.3 ± 5.6^3^
60 NaCl	379.5 ± 7.6^3^	463.1 ± 14.9^4^	842.6 ± 12.5^4^	69.0 ± 5.4^2^^3^	229.1 ± 5.9^4^	298.1 ± 4.3^4^
100 NaCl	276.3 ± 12.2^4^	398.3 ± 17.5^5^	674.6 ± 10.6^5^	58.0 ± 6.6^3^^4^	145.5 ± 8.0^5^	203.5 ± 5.2^5^
120 NaCl	243.2 ± 15.0^5^	394.6 ± 13.2^5^	637.8 ± 9.8^6^	49.0 ± 4.8^4^	122.1 ± 8.9^6^	171.1 ± 3.4^6^
0 CaCl_2_	536.7 ± 4.8^4^	634.0 ± 45.8^3^	1170.7 ± 27.1^3^	124.7 ± 5.9^3^	580.8 ± 17.9^1^	705.5 ± 8.9^2^
6.7 CaCl_2_	865.9 ± 6.5^1^	1383.4 ± 23.2^1^	2249.3 ± 21.5^1^	140.9 ± 6.2^2^	615.5 ± 15.9^1^	756.4 ± 9.6^1^
20 CaCl_2_	652.2 ± 7.4^2^	897.0 ± 15.3^2^	1549.2 ± 17.4^2^	172.9 ± 7.1^1^	530.0 ± 17.5^2^	702.9 ± 7.6^2^
40 CaCl_2_	588.0 ± 12.2^3^	596.5 ± 14.9^3^	1184.5 ± 12.6^3^	109.4 ± 6.4^4^	346.0 ± 12.0^3^	455.4 ± 6.4^3^
66 CaCl_2_	477.2 ± 8.6^5^	525.5 ± 21.1^4^	1002.7 ± 12.5^4^	92.4 ± 4.3^5^	240.1 ± 3.9^4^	332.5 ± 5.3^4^
80 CaCl_2_	465.0 ± 14.4^5^	473.2 ± 10.4^5^	938.2 ± 8.9^5^	73.5 ± 3.8^6^	213.5 ± 5.9^5^	287.0 ± 3.8^5^

**Notes.**

Values are mean ± SD (*n* = 4). Values in each column with the same number are not significantly different at *P* < 0.05, Tukey’s multiple test.

**Figure 1:**
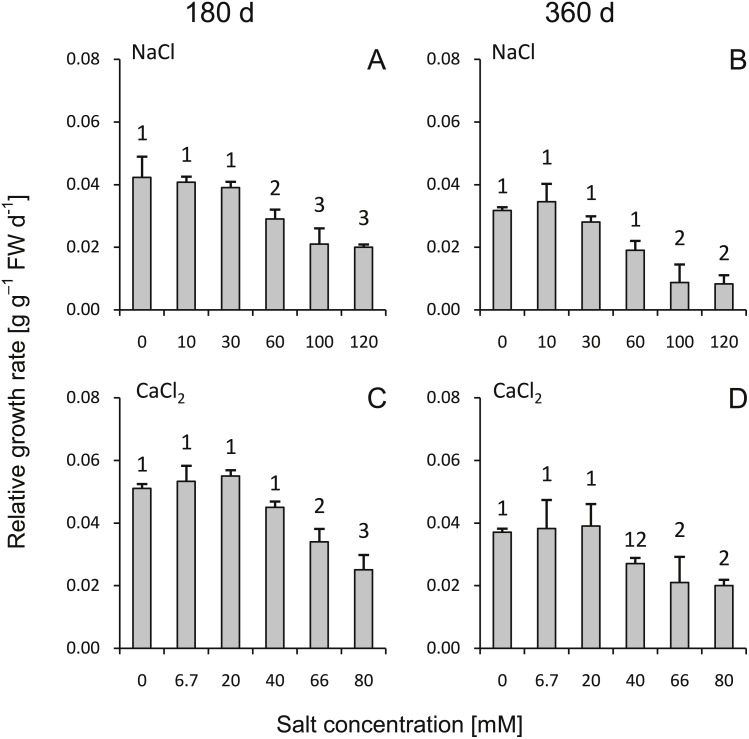
Effects of saline stress on the relative growth rate (RGR) of the silver maple seedlings exposed to different salinity levels (NaCl or CaCl_2_) after 180 (A, C) and 360 (B, D) days (g g^−1^ FW d^−1^). Values are mean ± SD (*n* = 4). Different numbers above each bar indicate significant differences by ANOVA followed by Tukey’s test at *P* < 0.05.

During the 180- to 360-d periods, the highest RGR was observed in the groups treated with low salinity (both NaCl and CaCl_2_, <30 mM and <20 mM, respectively) as well as with medium salt dose (60 mM NaCl and 40 mM CaCl_2_) (Fig. 1). The highest salinity substantially inhibited seedlings growth. Higher growth rates were usually observed during the 0- to 180-d period. Non saline controls and the groups subjected to the low salinity treatments were not significantly different from each other. Leaves of the plants from both NaCl and CaCl_2_ treatments (except control groups—0 mM NaCl and 0 mM CaCl_2_) developed a reddish color and became more brittle and dry; however, this was more prevalent for the NaCl-treated plants.

### Photosynthetic pigments

**Figure 2:**
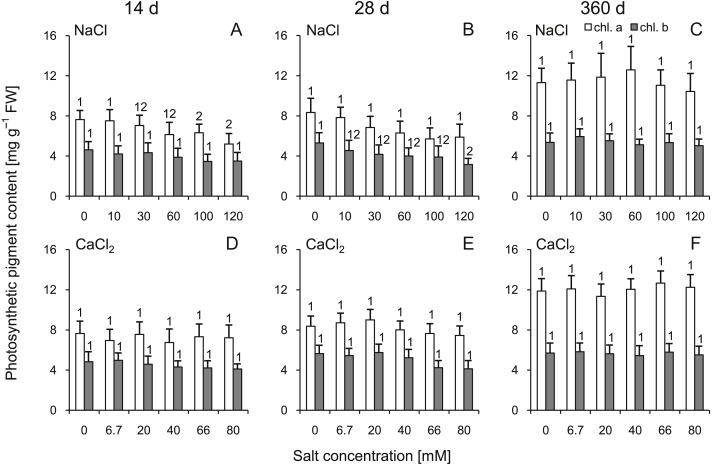
Effects of saline stress on the content of photosynthetic pigments (chlorophyll *a* and *b*) in the leaves from the silver maple seedlings exposed to different salinity levels (NaCl or CaCl_2_) after 14 (A, D), 28 (B, E) and 360 (C, F) days. Values are mean ± SD (*n* = 4). Different numbers above each bar indicate significant differences by ANOVA followed by Tukey’s test at *P* < 0.05.

Comparing to control groups, reduced concentrations of chlorophyll *a* were observed under the influence of solutions >100 mM NaCl at 14 d (Fig. 2). Significant reduction of chlorophyll *b* concentration was observed only at 28 d in group treated with 120 mM NaCl. CaCl_2_ solutions did not trigger any significant changes of chlorophyll *a* and *b* concentration in any group. At 360 d, the chlorophyll *a* and *b* contents were similar in all groups (no significant differences were observed for all corresponding NaCl vs CaCl_2_ comparisons; ANOVA with Tukey’s post-hoc test). Interestingly, this parameter were affected by all individual factors, while their interaction were not significant (Table S3).

### Proline content

**Figure 3:**
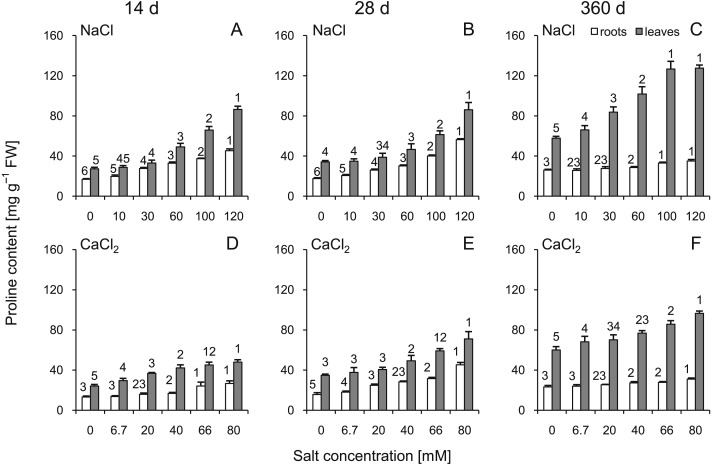
Effects of saline stress on the content of proline in the roots and leaves from the silver maple seedlings exposed to different salinity levels (NaCl or CaCl_2_) after 14 (A, D), 28 (B, E) and 360 (C, F) days. Values are mean ± SD (*n* = 4). Different numbers above each bar indicate significant differences by ANOVA followed by Tukey’s test at *P* < 0.05.

The proline content was significantly greater in the roots and leaves under all stress conditions (Fig. 3). The highest levels of proline compared to the control plants were observed at 360 d. In addition, greater changes were observed after treatment with high concentrations of NaCl than with CaCl_2_ (significant changes >60 mM NaCl in leaves and 100 mM in roots; ANOVA with Tukey’s post-hoc test). Both leaf and root proline amount were dependent of all tested factors and all their interactions (Tables S3 and S4).

### Superoxide dismutase activity

**Figure 4:**
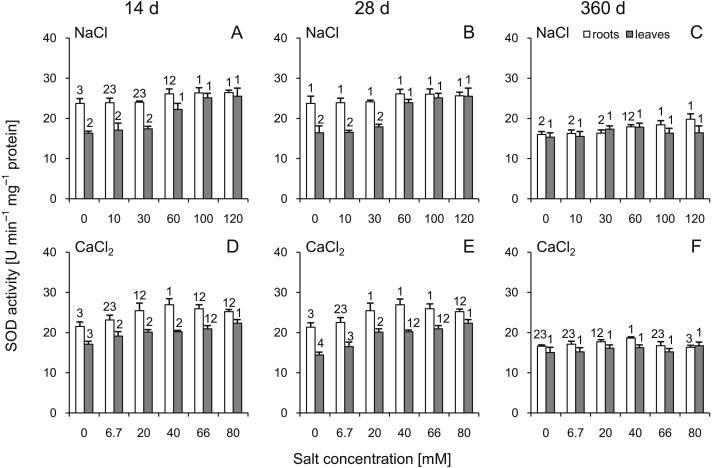
Effects of saline stress on the superoxide dismutase (SOD) activities in the roots and leaves from the silver maple seedlings exposed to different salinity levels (NaCl or CaCl_2_) after 14 (A, D), 28 (B, E) and 360 (C, F) days. Values are mean ± SD (*n* = 4). Different numbers above each bar indicate significant differences by ANOVA followed by Tukey’s test at *P* < 0.05.

A statistically significant increase in SOD activity was demonstrated in the roots and leaves at 14 d after treatment with NaCl as well as at 14 and 28 d after treatment with CaCl_2_ (Fig. 4). It is noteworthy that after 360 d of treatment, SOD activity in most cases did not differ from the control plants. The slight increase was recorded only in the roots of plants treated with 100 and 120 mM NaCl. Comparing corresponding salt treatments, some differences were observed in leaves (100 mM NaCl vs 66 mM CaCl_2_ at 14 d; 60 mM NaCl vs 40 mM CaCl_2_ and 100 mM NaCl vs 66 mM CaCl_2_ at 28 d) as well as in roots (only 120 mM NaCl vs 80 mM CaCl_2_ at 360). A three-way ANOVA showed significant influence of all factors, except interaction of time and salt treatment on activity of this enzyme in roots (Tables S3 and S4).

### Catalase activity

**Figure 5:**
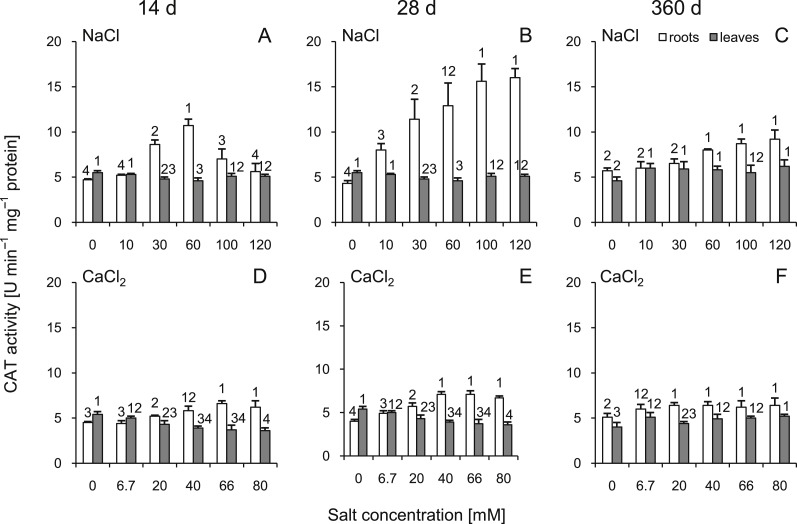
Effects of saline stress on the catalase (CAT) activities in the roots and leaves from the silver maple seedlings exposed to different salinity levels (NaCl or CaCl_2_) after 14 (A, D), 28 (B, E) and 360 (C, F) days. Values are mean ± SD (*n* = 4). Different numbers above each bar indicate significant differences by ANOVA followed by Tukey’s test at *P* < 0.05.

After 14 and 28 d, CAT activity significantly increased in the roots of almost all salt-treated plants (except 10 and 120 mM of NaCl at 14 d and 6.7 mM of CaCl_2_ at 14 and 28 d) (Fig. 5). At 360 d, this parameter was slightly but statistically significantly higher in both treatment variants. In the leaves, at 14 and 28 d after NaCl treatment, CAT activity decreased in groups treated with solutions of 30–60 mM. Significant differences were also observed in CaCl_2_ treated plants and those changes intensified with increasing salt concentration. At 360 d, slight but significant changes were observed. Interestingly, when compared to 14 and 28 d, the trend of changes reversed at 360 d (Fig. 5). Comparing corresponding salt treatments, differences were observed in roots (30 mM NaCl vs 20 mM CaCl_2_ and 60 mM NaCl vs 20 mM CaCl_2_ at 14 d; all comparisons at 28 d and 100 mM NaCl vs 66 mM CaCl_2_ and 120 mM NaCl vs 80 mM CaCl_2_ at 360 d) as well as in leaves (100 mM NaCl vs 66 mM CaCl_2_ and 120 mM NaCl vs 80 mM CaCl_2_ at 14 and 28 d and 30 mM NaCl vs 20 mM CaCl_2_ at 360 d). All factors showed significant influence on activity of CAT, except time and salt type interaction in leaves (three-way ANOVA) (Tables S3 and S4).

### Peroxidase activity

**Figure 6:**
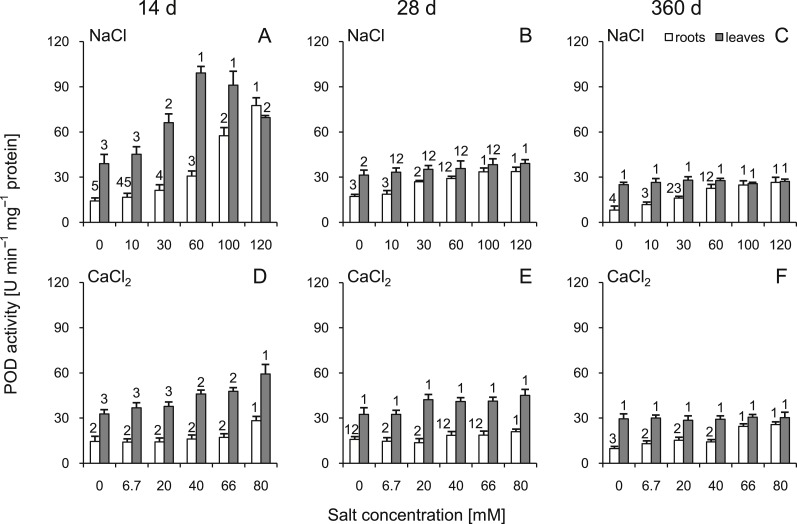
Effects of saline stress on the peroxidase (POD) activities in the roots and leaves from the silver maple seedlings exposed to different salinity levels (NaCl or CaCl_2_) after 14 (A, D), 28 (B, E) and 360 (C, F) days. Values are mean ± SD (*n* = 4). Different numbers above each bar indicate significant differences by ANOVA followed by Tukey’s test at *P* < 0.05.

A significant increase in POD activity was observed in the roots throughout the experiment. The highest POD activity (approximately five times greater than the control) was observed at 14 d in the plants treated with 120 mM NaCl solution (Fig. 6). In the leaves, POD activity increased with the increasing salt concentration at 14 and 28 d; however, greater changes were observed at 14 d. At 360 d after treatment, no significant differences in leaves were observed (Fig. 6). Comparing corresponding salt treatments, differences were observed leaves (all comparisons except control groups and the highest dose at 14 d) as well as in roots (30, 60, 100 mM NaCl vs 20, 40, 66 mM CaCl_2_, respectively, at 14 d, 30, 60, 100, 120 mM NaCl vs 20, 40, 66, 80 mM CaCl_2_, respectively, at 28 d, 30, 60, 120 mM NaCl vs 20, 40, 80 mM CaCl_2_, respectively, at 360 d). All factors showed significant influence on activity of POD (three-way ANOVA) (Tables S3 and S4).

### Protein content

In the roots watered with 100–120 mM NaCl solution, slight reductions in protein content were recorded in the early phase of the experiment (14 d), while a slight increase was characteristic of the 28 and 360 d. On the other hand, CaCl_2_ treatment caused an increase in the total protein content at 14, 28 and 360 d. Long-term changes in CaCl_2_-treated plants were similar to those observed in NaCl-treated plants. The leaves from plants subjected to CaCl_2_ treatment showed significantly increased protein content at 360 d (Table S1). A three-way ANOVA showed significant influence of all factors on protein content, both in roots and leaves (Tables S3 and S4).

## Discussion

Although the reduction in growth parameters were smaller in CaCl_2_-treated plants than in NaCl-treated plants, its harmful effect was still manifested. Hall, Hofstra & Lumis (1972) showed that increasing salt concentration (changing with distance from a de-iced highway) strongly affected the reduction of plant growth parameters, namely, the weight of buds, needle length and fresh weight, as well as annual radial increments in eastern white pine (*Pinus strobus* L.). It shows that injury caused by salt-based de-icing agents in woody species not tolerant to salt, such as *P. strobus* or studied *A. saccharinum* (Dirr, 1976), is a rather common result of road maintenance. Reduction of growth parameters (shoot length, total leaf area and total DW) was also reported for six olive cultivars treated with a relatively high dose of NaCl (>50 mM) (Chartzoulakis et al., 2002). The authors also indicated that a low dose of salt (25 mM) stimulated olive plant growth. Similar results were observed in our study for CaCl_2_ treatment (6.7 and 20 mM), while no positive effects of NaCl treatment were observed. Optimal calcium supplementation stimulates growth in many plant species (Fenn & Feagley, 1999). Furthermore, calcium is one of the macronutrients necessary for plants due to its role in cell development and stress response. It is also known to contribute to the physical integrity and functionality of membranes (Hopkins, 1995). It seems that a similar protective effect of low doses of calcium can be observed for silver maple.

It was demonstrated that snowmelting might be a substantial source of chloride ions and could increase the pH of soil, which resulted in its alkalization (Gałuszka et al., 2011). It was also shown that the alkaline pH of soils favored greater bioavailability of boron and reduced bioavailability of zinc for the examined trees. The negative symptoms included loss of photosynthetic activity and decreased vitality. Although in our study perlite was used as substratum (and alkalization was not monitored), such a mode of action is likely in natural conditions and probably enhance negative effect of salinity. Soil salinity causes rapid osmotic stress, which reduces growth of shoots, slows development and accelerates aging of cells in many plant species (Parida & Das, 2005). It is believed that Na^+^ causes osmotic stress in leaves, affects plant growth (reduces cell expansion and elongation), reduces leaf thickness and disturbs photosynthesis (Tester & Davenport, 2003). Application of salt was showed as agent reducing plant size and the number of leaves of cotton varieties (*Gossypium hirsutum* L.) (Saleh, 2012). The authors also showed that the number of leaves, chlorophyll *a* and *b* content and SPAD (soil plant analyses development) can be used to discriminate between salt-tolerant and salt-sensitive varieties. It was demonstrated that even plants not very susceptible to salinity (six species from the Leguminosae family) reacted to increasing NaCl concentration with a reduction in plant mass (Felker et al., 1981). Another study indicated that citrus (*Citrus tangerine* Hort. ex Tanaka) seedlings treated with 100 mM NaCl showed a reduction of leaf size, shoot and root length and seedling mass. Furthermore, photosynthesis limitation was observed (Wu, Zou & He, 2010). NaCl treatments (0, 50 and 100 mM) were also shown to decrease chlorophyll *a* and *b* contents, CO_2_ assimilation rate and stomatal conductance in *P. cathayana* and affected chloroplast functioning (Yang et al., 2009). Described perturbations affecting photosynthesis may have a negative impact on plant primary metabolism and plant growth. Our results showed that the reductions in chlorophyll *a* content under the influence of NaCl concentrations >60 mM at 14 d coincided with the reduction in RGR, FW and DW accumulation during the experiment, even at 360 d. It can be suggested that lowered chlorophyll content in the early phase of the experiment contributed to disturbances in plant metabolism that could not be fully compensated with time.

It is accepted that all trees are affected by salt stress, but some species are more tolerant than others (Dirr, 1976; Douglas, 2011). Numerous studies showed induced proline accumulation (Munns & Tester, 2008; Aziz, Martin-Tanguy & Larher, 1999; Acosta-Motos et al., 2017) during stress and indicated that it could be a protective mechanism against increased osmotic pressure resulting from salt stress (Hoque, 2008). Previous studies showed a high accumulation of proline in two poplar cultivars under the experimental conditions of salt stress (combined SO_2_ and NaCl treatment) (Karolewski, 1989). It was also indicated by Marin et al. (2010) that the highest concentration of proline in *in vitro*-grown roots of selected *Prunus* species could be observed in groups treated with the highest concentration of NaCl (180 mM), which is similar to the results provided in our study. Proline accumulation in roots may be one of the mechanisms involved in ROS scavenging and may contribute to the enzymatic quenching of ROS (Gill & Tuteja, 2010). This process is mediated by POD and CAT, whose activities increased significantly in salt-treated plants tested in our study at 14 and 28 d, respectively. Furthermore, elevated proline content was shown to protect enzymatic ROS-scavengers such as CAT and POD (Hoque et al., 2007). It can be concluded that coordination of proline accumulation and changing activities of CAT and POD may be treated as an adjustment to salt stress. On the other hand, our results indicate that for silver maple, such a mode of action could not be enough to cope with salt stress, which was manifested in plant growth parameters. Long-term accumulation of proline in leaves was shown in studies on olive (*Olea europaea* L.) treated with salt (Ben Rouina et al., 2006) and subjected to drought (BenAhmed et al., 2009). The authors suggested a possible beneficial role of proline in improving photosynthetic activity by triggering osmotic adjustment throughout the stress period. Similar results were also shown in our study regarding the coincidental increase in proline and stable chlorophyll contents.

Studies on two poplar species showed increased CAT and SOD activities in leaves and xylem sap of salt-stressed tolerant Euphrates poplar (*P. euphratica* Oliv.) and sensitive *P. popularis* ‘35–44’ plants, especially at high salinity levels (up to 250 mM NaCl). Separation of the isoforms of leaf SOD and CAT by polyacrylamide gel electrophoresis revealed that the salt-induced activities of CAT resulted from increased activity of all the detected isoenzymes (Wang et al., 2008). Particularly high SOD and CAT activities were found 18 d after NaCl treatment, which seems to be in agreement with our studies. The authors concluded that *P. euphratica* plants subjected to saline conditions controlled ROS homeostasis by osmotic control of NaCl-induced ROS production and by rapid upregulation of antioxidant defense to prevent oxidative damage (Wang et al., 2008). In studies on oak, SOD activity increased in young stalks under stress conditions (NaCl treatment). Moreover, the SOD isozyme pattern of oak leaves was altered when compared to the control group (Sehmer, Alaoui-Sosse & Dizengremel, 1995). The effects of different saline water irrigation levels were also studied in olive trees (*Olea europaea* L.) in which the activities of SOD and CAT in young leaves of salt-treated plants were 2.67 and 1.85 times higher than in the control, respectively. It was concluded that the interaction between the antioxidant defense system and proline content is involved in salt tolerance (BenAhmed et al., 2009). Similar results were shown in a study on physic nut (*Jatropha curcas* L.) (Silva et al., 2013). The conclusion can be formulated that a similar mechanism is mounted in silver maple subjected to salinity stress and that the activation of SOD is involved in enzymatic antioxidation, especially during intensifying salt stress (>30 mM of NaCl).

We showed that the activity of CAT increased after NaCl and CaCl_2_ treatment. This increase was marked in the roots at 14 and 28 d after NaCl treatment, while we were not able to detect any greater changes in the leaves during the experiment. In the contrary, studies conducted on salt-resistant brush cherry (*Eugenia myrtifolia* Sims) watered with saline reclaimed water showed that long-term response (23 weeks) involved increased CAT activity in leaves, but this change seemed not be correlated with salt dose (Acosta-Motos et al., 2017). The authors pointed that the observed CAT changes did not appear to be enough to cope with the stress induced by the long-term exposure to salinity. Based on previous studies of barley (*Hordeum vulgare* L.) cultivars (Fan et al., 2014), it was concluded that changes in CAT activity in leaves should not be used as a standalone biochemical marker proving salinity tolerance in crop plants. We showed that sequential biochemical testing of roots and leaves accompanied with growth analysis can fulfill the requirements for salt sensitivity testing in trees. CAT was recently suggested as a main factor controlling salt tolerance triggered by H_2_O_2_ (Gondim et al., 2012). This is in agreement with experiments conducted on physic nut (*Jatropha curcas* L.) which is a salt-tolerant species (Gao et al., 2008). It seems that elevated CAT activity in both leaves and roots has a protective effect, while increased activity just in one organ is not enough to trigger adequate response. Surprisingly, in the early stage of our experiments, CAT activity remarkably increased just in the groups treated with the medium NaCl solutions (30 and 60 mM). Increasing concentrations of salt (100 and 120 mM) stopped this effect, which may indicate that plants were not able to cope with severe salt stress. A similar relationship was recorded by Sorkheh et al. (2012) for 8 wild almond species (*Prunus* spp.) treated with a wide range of NaCl solutions. The gradual increase in CAT activity after 28 d during NaCl-induced stress recorded in the present study is similar to the phenomenon reported before in date palm (*Phoenix dactylifera* L.) (Al-Qurainy et al., 2017). It is noteworthy that CaCl_2_ triggered only slight CAT changes in silver maple tissues, which may indicate its lower phytotoxicity.

We observed that POD activity increased dramatically in the roots at 14 d after treatment with NaCl and then decreased with time. Moreover, a positive relationship between POD activity level and salt concentration was observed in both NaCl and CaCl_2_. The profiles of changes were similar; however, they were markedly more pronounced in the NaCl variant. Some studies indicate that POD is involved in a salinity stress response in Kashgar tamarisk (*Tamarix hispida* Willd.) and is regulated by ABA-dependent signaling pathways covering salt tolerance in many plant species (Gao et al., 2010). Due to the multifunctional activity of POD, it is believed that its activity is a good marker of plant stress response; however, its role during abiotic stress is still not fully elucidated (Passardi et al., 2005). It was also proposed that peroxidases may play a protective role acting as scavengers of H_2_O_2_, which overproduction took place under salt stress (Novo-Uzal et al., 2014). No greater changes in the leaves and roots were observed 360 d after treatment. Therefore, it can be concerned that one year is enough to cope with salt stress and to start recovery. On the other hand, long-lasting stress under urban conditions is severe, and its cyclicality may make the plant unable to cope with it.

## Conclusion

It was shown in this study that the plant growth parameters, namely, fresh and dry weight, were reduced by high concentrations of NaCl and CaCl_2_. Furthermore, the response strictly depended on the salt type and dose. Our experiments indicate the existence of short-term stress-coping reactions (increased enzyme activity coincident with increased proline content under salt stress). Restabilization of long-term biochemical traits and inhibited growth suggest that the studied species can survive de-icing treatments, but subsequent recovery is needed. Overall, this indicates that silver maple seedlings should be considered susceptible to long-lasting severe salt stress. Hence, it is recommended to use silver maple plantings on secondary avenues or park alleys, while planting on often de-iced pavements and roads (e.g., highways) should be avoided. Our study also suggests that CaCl_2_ shows less toxicity to plants and therefore that its use should be considered.

##  Supplemental Information

10.7717/peerj.5958/supp-1 Table S1 Effects of saline stress on the protein content in the roots and leaves from the silver maple seedlings exposed to different salinity levels (NaCl or CaCl_2_) after 14, 28 and 360 days [mg g^−1^FW] Values are mean ± SD (*n* = 4). Different lower-case letters indicate significant differences by ANOVA followed by Tukey’s test at *P* < 0.05.

10.7717/peerj.5958/supp-2 Table S2 Results of three-way ANOVA examining the effects of the studied factors on growth parameters of silver maple (*Acer saccharinum* L.) n.s. –not significant.

10.7717/peerj.5958/supp-3 Table S3 Results of three-way ANOVA examining the effects of the studied factors on biochemical parameters in leaves of silver maple (*Acer saccharinum* L.) n.s. –not significant.

10.7717/peerj.5958/supp-4 Table S4 Results of three-way ANOVA examining the effects of the studied factors on biochemical parameters in roots of silver maple (Acer saccharinum L.) n.s. –not significant.

10.7717/peerj.5958/supp-5 Figure S1 Values of relative growth rate measured in this study Each data point indicates value of relative growth rate [g g^−1^ FW d^−1^].

10.7717/peerj.5958/supp-6 Figure S2 Values of chlorophyll content measured in this study Each data point indicates chlorophyll content [mg g^−1^FW].

10.7717/peerj.5958/supp-7 Figure S3 Values of proline content measured in this study Each data point indicates proline content [mg g^−1^ FW).

10.7717/peerj.5958/supp-8 Figure S4 Values of SOD activity measured in this study Each data point indicates enzymatic activity [U min^−1^mg protein^−1^].

10.7717/peerj.5958/supp-9 Figure S5 Values of CAT activity measured in this study Each data point indicates enzymatic activity [U min^−1^mg protein^−1^].

10.7717/peerj.5958/supp-10 Figure S6 Values of POD activity measured in this study Each data point indicates enzymatic activity [U min^−1^ mg protein^−1^].

10.7717/peerj.5958/supp-11 Table S5 Values of fresh and dry weight accumulation measured in this study Each data point indicates value of selected weight parameter [mg plant^−1^].

10.7717/peerj.5958/supp-12 Table S6 Values of protein content measured in this study Each data point indicates protein content [mg g^−1^FW].

## Additional Information and Declarations

### Data Availability

The following information was supplied regarding data availability: The raw data are provided in the Supplemental Files.
