# Supplementary material for: Biochemical and growth responses of silver maple (Acer saccharinum L.) to sodium chloride and calcium chloride
Source: PeerJ. 2018 Dec 21;6:e5958. doi: 10.7717/peerj.5958 (PMC6309728; doi:10.7717/peerj.5958)
Supplement: Table S1 — Values are mean ± SD (n = 4). Different lower-case letters indicate significant differences by ANOVA followed by Tukey’s test at P < 0.05. [file peerj-06-5958-s001.docx]

**Supplemental table 1 Effects of saline stress on the protein content in the roots and leaves from the silver maple seedlings exposed to different salinity levels (NaCl or CaCl_2_) after 14, 28 and 360 days [mg g^-1^ FW].** Values are mean ± SD (n = 4). Different lower-case letters indicate significant differences by ANOVA followed by Tukey's test at P < 0.05.

| Salinity (mM) | 14 days | 28 days | 360 days |
| --- | --- | --- | --- |
| roots | | | |
| 0 NaCl | 4.4 ± 0.1ab | 4.4 ± 0.1d | 4.7 ± 0.2c |
| 10 NaCl | 4.7 ± 0.3ab | 5.0 ± 0.1c | 5.0 ± 0.3bc |
| 30 NaCl | 5.2 ± 0.5ab | 5.9 ± 0.2b | 5.3 ± 0.2ab |
| 60 NaCl | 5.3 ± 0.4a | 7.7 ± 0.8a | 5.5 ± 0.2ab |
| 100 NaCl | 4.2 ± 0.5ab | 6.3 ± 0.4b | 5.1 ± 0.3bc |
| 120 NaCl | 3.7 ± 0.7b | 6.1 ± 0.1b | 5.8 ± 0.3a |
|  |  |  |  |
| 0 CaCl_2_ | 4.8 ± 0.3b | 4.9 ± 0.4d | 4.4 ± 0.2b |
| 6.7 CaCl_2_ | 5.1 ± 0.2b | 4.9 ± 0.1cd | 4.8 ± 0.4ab |
| 20 CaCl_2_ | 6.0 ± 0.5a | 5.4 ± 0.1bcd | 4.8 ± 0.3ab |
| 40 CaCl_2_ | 6.1 ± 0.3a | 5.5 ± 0.4bc | 5.0 ± 0.5ab |
| 66 CaCl_2_ | 6.2 ± 0.6a | 5.7 ± 0.3b | 5.2 ± 0.4ab |
| 80 CaCl_2_ | 6.4 ± 0.4a | 6.4 ± 0.3a | 5.0 ± 0.3a |
| leaves | | | |
| 0 NaCl | 12.2 ± 0.7ab | 11.6 ± 0.7c | 14.4 ± 0.6a |
| 10 NaCl | 12.9 ± 0.8a | 12.4 ± 0.9bc | 14.5 ± 0.9a |
| 30 NaCl | 13.4 ± 0.6a | 14.7 ± 0.9a | 14.6 ± 0.8a |
| 60 NaCl | 11.0 ± 0.8bc | 14.2 ± 0.6ab | 14.8 ± 0.4a |
| 100 NaCl | 9.7 ± 0.7cd | 13.9 ± 0.9ab | 14.6 ± 1.1a |
| 120 NaCl | 9.1 ± 0.6d | 14.1 ± 1.1ab | 14.8 ± 0.6a |
|  |  |  |  |
| 0 CaCl_2_ | 12.5 ± 0.8d | 13.9 ± 1.0c | 13.7 ± 0.5b |
| 6.7 CaCl2 | 12.9 ± 0.9cd | 15.5 ± 0.2b | 14.0 ± 0.9b |
| 20 CaCl_2_ | 14.2 ± 0.9bc | 16.2 ± 0.4ab | 16.1 ± 0.5a |
| 40 CaCl_2_ | 15.2 ± 0.7ab | 15.9 ± 0.7ab | 16.5 ± 0.6a |
| 66 CaCl_2_ | 16.0 ± 0.4a | 16.9 ± 0.3a | 16.1 ± 0.6a |
| 80 CaCl_2_ | 16.2 ± 0.6a | 16.9 ± 0.4a | 17.0 ± 0.4a |
