# Supplementary material for: Biochemical and growth responses of silver maple (Acer saccharinum L.) to sodium chloride and calcium chloride
Source: PeerJ. 2018 Dec 21;6:e5958. doi: 10.7717/peerj.5958 (PMC6309728; doi:10.7717/peerj.5958)
Supplement: Table S2 — n.s. –not significant. [file peerj-06-5958-s002.docx]

**Supplemental Table 2. Results of three-way ANOVA examining the effects of the studied factors on growth parameters of silver maple (*Acer saccharinum* L.).** n.s. – not significant

| Parameter | Factor | df | F | p |
| --- | --- | --- | --- | --- |
| RGR | T (Time) | 1 | 100.9 | <0.001 |
|  | S (Salt type) | 1 | 78.0 | <0.001 |
|  | C (Salt concentration) | 5 | 54.8 | <0.001 |
|  | T x S | 1 | 2.8 | n.s. |
|  | T x C | 5 | 3.2 | 0.05 |
|  | S x C | 5 | 4.2 | 0.01 |
|  | T x S x C | 5 | 2.7 | 0.05 |
| FW root | T | 1 | 697.0 | <0.001 |
|  | S | 1 | 5232.0 | <0.001 |
|  | C | 5 | 1137.0 | <0.001 |
|  | T x S | 1 | 8.0 | 0.01 |
|  | T x C | 5 | 38.0 | <0.001 |
|  | S x C | 5 | 240.0 | <0.001 |
|  | T x S x C | 5 | 23.0 | <0.001 |
| FW shoot | T | 1 | 9688.0 | <0.001 |
|  | S | 1 | 2759.0 | <0.001 |
|  | C | 5 | 750.0 | <0.001 |
|  | T x S | 1 | 148.0 | <0.001 |
|  | T x C | 5 | 183.0 | <0.001 |
|  | S x C | 5 | 58.0 | <0.001 |
|  | T x S x C | 5 | 49.0 | <0.001 |
| FW total | T | 1 | 12693.0 | <0.001 |
|  | S | 1 | 11251.0 | <0.001 |
|  | C | 5 | 2955.0 | <0.001 |
|  | T x S | 1 | 119.0 | <0.001 |
|  | T x C | 5 | 293.0 | <0.001 |
|  | S x C | 5 | 156.0 | <0.001 |
|  | T x S x C | 5 | 111.0 | <0.001 |
| DW root | T | 1 | 263.7 | <0.001 |
|  | S | 1 | 863.9 | <0.001 |
|  | C | 5 | 105.9 | <0.001 |
|  | T x S | 1 | 10.5 | 0.01 |
|  | T x C | 5 | 4.8 | <0.001 |
|  | S x C | 5 | 18.2 | <0.001 |
|  | T x S x C | 5 | 1.0 | n.s. |
| DW shoot | T | 1 | 4837.0 | <0.001 |
|  | S | 1 | 1834.0 | <0.001 |
|  | C | 5 | 1837.0 | <0.001 |
|  | T x S | 1 | 153.0 | <0.001 |
|  | T x C | 5 | 106.0 | <0.001 |
|  | S x C | 5 | 8.0 | <0.001 |
|  | T x S x C | 5 | 4.0 | 0.01 |
| DW total | T | 1 | 13436.0 | <0.001 |
|  | S | 1 | 7926.0 | <0.001 |
|  | C | 5 | 5065.0 | <0.001 |
|  | T x S | 1 | 387.0 | <0.001 |
|  | T x C | 5 | 313.0 | <0.001 |
|  | S x C | 5 | 22.0 | <0.001 |
|  | T x S x C | 5 | 13.0 | <0.001 |
