# Supplementary material for: Biochemical and growth responses of silver maple (Acer saccharinum L.) to sodium chloride and calcium chloride
Source: PeerJ. 2018 Dec 21;6:e5958. doi: 10.7717/peerj.5958 (PMC6309728; doi:10.7717/peerj.5958)
Supplement: Table S3 — n.s. –not significant. [file peerj-06-5958-s003.docx]

**Supplemental Table 3. Results of three-way ANOVA examining the effects of the studied factors on biochemical parameters in leaves of silver maple (*Acer saccharinum* L.).** n.s. – not significant

| Parameter | Factor | df | F | p |
| --- | --- | --- | --- | --- |
| Chlorophyll a | T (Time) | 2 | 170.9 | <0.001 |
|  | S (Salt type) | 1 | 20.7 | <0.001 |
|  | C (Salt concentration) | 5 | 4.1 | 0.01 |
|  | T x S | 2 | 2.8 | n.s. |
|  | T x C | 10 | 1.4 | n.s. |
|  | S x C | 5 | 2.1 | n.s. |
|  | T x S x C | 10 | 0.6 | n.s. |
| Chlorophyll b | T | 2 | 28.7 | <0.001 |
|  | S | 1 | 17.6 | <0.001 |
|  | C | 5 | 6.0 | <0.001 |
|  | T x S | 2 | 2.3 | n.s. |
|  | T x C | 10 | 1.2 | n.s. |
|  | S x C | 5 | 0.3 | n.s. |
|  | T x S x C | 10 | 0.4 | n.s. |
| Proline | T | 2 | 1486.1 | <0.001 |
|  | S | 1 | 114.5 | <0.001 |
|  | C | 5 | 496.8 | <0.001 |
|  | T x S | 2 | 26.8 | <0.001 |
|  | T x C | 10 | 9.9 | <0.001 |
|  | S x C | 5 | 32.9 | <0.001 |
|  | T x S x C | 10 | 7.2 | <0.001 |
| SOD | T | 2 | 217.9 | <0.001 |
|  | S | 1 | 24.0 | <0.001 |
|  | C | 5 | 99.6 | <0.001 |
|  | T x S | 2 | 4.0 | 0.05 |
|  | T x C | 10 | 16.4 | <0.001 |
|  | S x C | 5 | 12.2 | <0.001 |
|  | T x S x C | 10 | 4.1 | <0.001 |
| CAT | T | 2 | 35.6 | <0.001 |
|  | S | 1 | 206.3 | <0.001 |
|  | C | 5 | 14.7 | <0.001 |
|  | T x S | 2 | 0.00 | n.s. |
|  | T x C | 10 | 18.0 | <0.001 |
|  | S x C | 5 | 9.2 | <0.001 |
|  | T x S x C | 10 | 3.8 | <0.001 |
| POX | T | 2 | 634.0 | <0.001 |
|  | S | 1 | 28.9 | <0.001 |
|  | C | 5 | 53.0 | <0.001 |
|  | T x S | 2 | 149.4 | <0.001 |
|  | T x C | 10 | 18.9 | <0.001 |
|  | S x C | 5 | 5.8 | <0.001 |
|  | T x S x C | 10 | 7.6 | <0.001 |
| Protein | T | 2 | 138.6 | <0.001 |
|  | S | 1 | 329.2 | <0.001 |
|  | C | 5 | 20.4 | <0.001 |
|  | T x S | 2 | 39.1 | <0.001 |
|  | T x C | 10 | 6.0 | <0.001 |
|  | S x C | 5 | 25.5 | <0.001 |
|  | T x S x C | 10 | 14.6 | <0.001 |
