# Supplementary material for: Biochemical and growth responses of silver maple (Acer saccharinum L.) to sodium chloride and calcium chloride
Source: PeerJ. 2018 Dec 21;6:e5958. doi: 10.7717/peerj.5958 (PMC6309728; doi:10.7717/peerj.5958)
Supplement: Table S4 — n.s. –not significant. [file peerj-06-5958-s004.pdf]

Supplemental Table 4. Results of three-way ANOVA examining the effects of the studied factors on biochemical parameters in roots of silver maple (*Acer saccharinum* L.). n.s. – not significant

Parameter	Factor	df	F	p
Proline	T	2	213.7	<0.001
	S	1	592.7	<0.001
	C	5	583.7	<0.001
	T x S	2	147.5	<0.001
	T x C	10	57.5	<0.001
	S x C	5	5.4	<0.001
	T x S x C	10	6.6	<0.001
SOD	T	2	947.7	<0.001
	S	1	4.2	0.05
	C	5	35.9	<0.001
	T x S	2	0.1	n.s.
	T x C	10	0.9	n.s.
	S x C	5	8.5	<0.001
	T x S x C	10	4.0	<0.001
CAT	T	2	117.6	<0.001
	S	1	546.0	<0.001
	C	5	175.6	<0.001
	T x S	2	87.1	<0.001
	T x C	10	24.9	<0.001
	S x C	5	19.2	<0.001
	T x S x C	10	17.7	<0.001
POX	T	2	67.0	<0.001
	S	1	231.5	<0.001
	C	5	164.6	<0.001
	T x S	2	49.8	<0.001
	T x C	10	11.9	<0.001
	S x C	5	21.7	<0.001
	T x S x C	10	6.8	<0.001
Protein	T	2	37.6	<0.001
	S	1	7.6	0.01
	C	5	35.5	<0.001
	T x S	2	80.5	<0.001
	T x C	10	6.5	<0.001
	S x C	5	9.9	<0.001
	T x S x C	10	9.2	<0.001
